# Editorial: Novel Anti-Inflammatory Approaches for Cystic Fibrosis Lung Disease: Identification of Molecular Targets and Design of Innovative Therapies

**DOI:** 10.3389/fphar.2021.794854

**Published:** 2021-11-04

**Authors:** Carla Maria Pedrosa Ribeiro, Noel Gerard McElvaney, Giulio Cabrini

**Affiliations:** ^1^ Marsico Lung Institute/Cystic Fibrosis Research Center, Department of Medicine, Department of Cell Biology and Physiology, The University of North Carolina at Chapel Hill, Chapel Hill, NC, United States; ^2^ Irish Centre for Genetic Lung Disease, Royal College of Surgeons in Ireland, Dublin, Ireland; ^3^ Department of Life Sciences and Biotechnology, and Center on Innovative Therapies for Cystic Fibrosis, University of Ferrara, Ferrara, Italy; ^4^ Department of Neurosciences, Biomedicine and Movement, University of Verona, Verona, Italy

**Keywords:** cystic fibrosis, cystic fibrosis transmembrane conductance regulator, lung, inflammation, infection, modulators, therapeutic approach, drug development

Cystic Fibrosis (CF) is a monogenic disease caused by mutations of the Cystic Fibrosis (CF) Transmembrane conductance Regulator (*CFTR*) gene encoding a chloride and bicarbonate transporter (Kerem et al., 1989; Riordan et al., 1989; Rommens et al., 1989). CF disease affects different organs, with the chronic lung pathology being the main cause of morbidity and reduction of life expectancy of these patients (Shteinberg et al., 2021). Pulmonary disease has been demonstrated since early infancy and progresses with airways obstruction and bronchial damages, representing the major challenge for the cure of these patients (Stoltz et al., 2015). Pathophysiology of CF lung disease is still a matter of debate (Bergeron and Cantin, 2019); one leading hypothesis is that the defective CFTR ion channel activity promotes dehydration of the airway surface liquid, alters mucus properties, and decreases mucociliary clearance, favoring the onset of inflammation together with recurrent and, ultimately, chronic bacterial infection (Boucher, 2019; Esther et al., 2019).

The immune response of CF lungs is characterized by exaggerated inflammation, abundant pro-inflammatory cytokines and chemokines in the bronchial mucosa, and massive lumenal infiltrates of polymorphonuclear neutrophils, which release proteases and reactive oxygen species. This overall process leads to chronic airway obstruction and pulmonary damage (Roesch et al., 2018). The contribution of cellular components (e.g., bronchial epithelial cells, neutrophils, lymphocytes, macrophages, dendritic cells), subcellular organelles (e.g., endoplasmic reticulum, mitochondria), intracellular signalling pathways (e.g., MAP kinases, calcium signaling, the unfolded protein response) and transcription factors (e.g., NF-κB and XBP-1) has been investigated regarding the pathophysiology of the exaggerated CF lung inflammation. The relevance of infection and the role of mutant CFTR protein in this complex interplay have also been explored (Roesch et al., 2018).

Although anti-inflammatory corticosteroids and ibuprofen have shown limited efficacy in CF patients, the development of novel drugs for CF lung inflammation has been neglected for many years. Instead, drug discovery of the so-termed modulators, aimed at correcting the trafficking and function of mutant CFTR, became a priority. Nevertheless, it remains to be established whether CFTR modulators will be sufficient to halt the inflammatory processes and mucus overproduction responsible for lung damage in CF adolescents and adults with advanced lung disease. In addition, it is not known whether modulators will be capable of preventing the establishment of lung infection, inflammation and mucus overproduction when administered to CF infants exhibiting early signs of pulmonary disease. Thus, the development of more effective “CF tailored” anti-inflammatory drugs, that can be combined with therapeutic protocols utilizing novel CFTR modulators and antibacterial drugs, remains an unmet need that requires further research (Roesch et al., 2018).

The 20 articles collected in this Research Topic focus on the most recent advances on CF lung inflammation, mainly on novel molecular targets and on innovative pharmacological approaches. In a review of the literature, Mitri et al. show how the topic of CF lung inflammation has been partially neglected in respect to the compelling priority of discovering and developing effective CFTR modulators. They recall the main issues of the debate on CF lung inflammation, starting from the pathophysiology of inflammation and infection, the alteration of the airway mucus, the role of proteases released from polymorphonuclear neutrophils, and the lipid dysregulation. They recapitulate the limitations of the current anti-inflammatory drugs and describe a series of recent innovative therapeutic approaches proposed by different researchers. The view that research on anti-inflammatory approaches should not remain the “poor relative” of the CF drug discovery pipelines has been further strengthened by Kunzi et al., who described the evolution of the CF lung pathophysiology in the aging CF population, where the median predicted survival is now over 40 years, with several implications on cellular senescence, stem cell exhaustion, altered inter-cellular communications, loss of proteostasis, and mitochondrial dysfunction.

Recurrent infections and persistent inflammation in CF slowly and progressively worsen lung function. Since the CF anti-inflammatory research has moved its first steps from pre-clinical to clinical investigation, it is crucial to properly evaluate the effectiveness of new drugs and the advancements in the field. Perrem and Ratjen examined the most informative biomarkers of clinical progression and endpoints that can be used to test the efficacy of drugs, which are key for successful clinical trials. Moreover, considering that neutrophils and their products can be targets of anti-inflammatory drugs in CF (Torphy et al., 2015), these authors renewed the discussion regarding reducing the damaging effects of inflammation while maintaining effective immunological defenses against bacterial infection, a lesson learned from the early clinical trials.

Identification of molecular targets faces the obstacle of redundant intracellular regulatory pathways, one of the main features of the lung immune responses. Although redundancy is a beneficial evolutionary feature aimed to effectively fight infective offense, this has been a challenging issue in the search for effective anti-inflammatory targets that could be frequently bypassed by alternative intracellular signals. From a mechanistic point of view, this research field followed different directions, e.g., identification of CF immune defects that can be circumvented, identification of critical signaling pathways of the initial innate immune response elicited in bronchial epithelial cells, targeting neutrophils and phagocytes as the main effectors of the inflammatory response, and activation of the pathways involved in resolution of inflammatory processes.

Studies with pathogen-host interactions involving phagocytes have indicated that the CF immune system is inefficient at combating bacterial infections. Here, van Heeckeren et al. demonstrate the immune-defective role of CF macrophages by trasferring hematopoietic and mesenchymal stem cells between *CFTR*-deficient and *CFTR* wild type murine models. Their approach reproduced or restored anti-infective and anti-inflammatory functions, opening an experimental path towards cell-based therapies for CF. In a parallel article, Di Pietro et al. dissected the reduced bactericidal capacity of CF macrophages, demonstrating a defect in the Heme Oxygenase-1/Carbon Monoxide pathway and proposing an experimental approach to restore the bactericidal activity in these cells. Analysis of defective bacterial killing of CF phagocytes has been extended from macrophages to neutrophils. Cantin et al. showed that the intracellular killing of Gram-negative bacteria by CF neutrophils is inhibited by CF airway mucins that protect the engulfed *Pseudomonas aeruginosa* by inhibiting respiratory burst and lysozyme-mediated bacterial killing. McQuillan et al. studied an additional aspect of bacterium-phagocyte interactions by investigating the anti-neutrophil cytoplasmic autoantibodies (ANCA) directed towards the bactericidal permeability increasing (BPI) protein, the latter acting as anti-bacterial weapon released by neutrophilic granules. Whereas high concentrations of BPI are present in the bronchoalveolar lavage fluid of CF patients, a parallel increase of ANCA complexed with BPI was shown to be a key mechanism for reducing the overall bactericidal capacity of the CF innate immune system. On the other hand, the studies of Lee et al. focused on host-bacteria interactions by reviewing the emerging aspects of the CF microbiote and microbiome. These authors suggested the use of multi-omics technologies to expand the understanding of basis for the inneficient CF immune system.

The bronchial epitelium has been considered for long time a simple physical barrier to protect the airway mucosa from the penetration of infectious agents, dust and pollution. This vision drastically changed in recent years, where the bronchial pseudostratified epithelium has gained relevance both as a key contributor of innate immune responses (e.g., *via* cytokine production) and in cross-talking with “professional” immune cells, such as macrophages, neutrophils, lymphocytes, and dendritic cells (for a review on bronchial epithelium, see Herr et al., 2020). Thus, the intracellular signaling evoked in bronchial epithelial cells by *Pseudomonas aeruginosa*, *Staphylococcus aureus*, other microrganisms and their products affecting CF patients has been investigated to identify critical organelles and pathways that can be modulated by novel anti-inflammatory drugs. Here, Rimessi et al. reviewed several articles dealing with regulation of intracellular calcium, a key second messenger altered in CF bronchial epithelial cells either as a result of the basic CFTR protein defect or the chronic exposure to colonizing bacteria. These authors reviewed molecules under pre-clinical investigation aimed to modify the dysregulated pathways. In parallel, Patergnani et al. recall the effect of infectious agents on mitochondria of CF bronchial epithelial cells, which become targets of the intracellular signaling resulting from activation of Toll-like Receptors. Via increased intracellular calcium fluxes through specific mitochondrial calcium channels, mitochondria actively contribute for the production of intracellular reactive oxygen species and the activation of the inflammasome. An additional set of data involving lung epithelia was contributed by Lin et al. They investigated intracellular signaling in a cell line of epithelial pneumocytes exposed to bacterial endotoxin, and found a protective role of the Clara Cell secretory protein (CC16) against apoptosis.

While the majority of approaches in search of the best molecular targets for anti-inflammatory molecules has been aimed to downmodulate the activation phase of the innate immune response, recent efforts have also been directed towards the upregulation of physiological pathways that participate in the resolution of inflammation. Briottet et al. and Recchiuti et al. reviewed specialized pro-resolvin mediators of inflammation, such as lipoxins, resolvins, protectins, and marensins, and the underlying specific dysregulations found in different CF cell models. They suggested that a compensatory or regulatory action can be a novel anti-inflammatory approach for CF lung disease.

Exploring the efficacy, the mechanisms of action, or the delivery strategies of new molecules aimed to control lung inflammation in preclinical models has been also presented in this article collection. Voynow et al. evaluated the feasibility of testing inhaled glycosaminoglycans, such as modified heparins devoid of anticoagulant effects, as novel anti-elastase allosteric inhibitors aimed at intervening against a major effector of CF bronchial damage released by airway neutrophils. Yougbare et al. focused on phosphodiesterase isoform 4 (PDE4), a regulator of chronic inflammation, by testing the effect of the PDE4 inhibitor NCS 613. Zhang et al. presented the protective effect of pterostilbene, a stilbene derivative originally isolated from medicinal plants, in a model of murine acute lung inflammation induced by endotoxin. They showed that the protective action of pterostilbene was mediated by activation of the anti-oxidant transcription factor Nrf2, the enzyme HO-1, and inhibition of NF-κB. In a similar murine lung model of acute inflammation, Jin et al. utilized nasal delivery of the anti-inflammatory flavonoid molecule hesperidin complexed with chitosan nanoparticles and showed the efficacy of this nasal delivery system to suppress the airways’ cytokine storm evoked by endotoxin.

Correction of the basic CFTR ion transport defect by effective CFTR modulators from early infancy might be beneficial against bacterial infection and inflammation. This key issue is being investigated in both preclinical models and clinical trials. Bene et al. focused on human epididymis protein 4 (HE4), whose plasma or serum concentrations associate with the degree of CF lung disease, reflecting its potential role as biomarker for CF lung inflammation. These authors found that CFTR modulators partially corrected mutant CFTR and reduced pro-inflammatory challenge-induced HE4 expression, suggesting a potential link between CFTR rescue and reduction of inflammation. In parallel, Hisert et al. investigated the genome-wide transcriptome in blood monocytes from CF patients compound heterozygotes with the gating R117H-CFTR mutation, before and after 7 days of clinical trial with the CFTR potentiator Ivacaftor (VX-770). They found that 7 days of Ivacaftor increased the expression of 50 genes involved in pro-inflammatory pathways, and increased the plasma levels of the monocyte chemokines CCL2 (MCP-1) and CXCL2 (MIP-2), suggesting an immune regulatory effect of this CFTR modulator.

Notably, little is known whether the lung inflammatory milieu influences the efficacy of CFTR modulators. Gentzsch et al. addressed this question utilizing a model consisting of exposing fully differentiated primary F508del homozygous bronchial epithelia to supernatant from mucopurulent material (SMM) harvested from the airways of excised lungs from CF patients. While the authors did not find an anti-inflammatory effect of various combinations of CFTR modulators, based on the release of CXCL8 (IL-8), they showed that SMM augmented the rescue of F508del-CFTR by the modulators. Although Gentzsch et al. did not suggest to increase CF airway inflammation to enhance CFTR rescue, understanding the mechanisms underlying these findings may lead to improved CFTR modulator therapies.

In summary, this Research Topic provided a platform for CF researchers to present novel findings on therapeutic targets for CF inflammation and to learn new insights on the interplay between airway inflammation and enhanced efficacy of CFTR modulators. Novel therapeutic strategies are needed to reduce the detrimental effects of chronic inflammation without blunting the immune defenses against infection. Additional research is necessary to understand how the efficiency of CFTR modulators is increased by airway inflammation. These issues represent unmet needs for CF patients that should be no longer neglected.
